# Navigation-Based Telehealth Informed Decision-Making for Prostate Cancer Screening in Black Men

**DOI:** 10.3390/curroncol31070273

**Published:** 2024-06-28

**Authors:** Djibril M. Ba, Chrispin Kayembe, Joe Littlejohn, Lauren J. Van Scoy, Erika VanDyke, James Williams, Avnish Katoch, Neil C. Shook, Yue Zhang, Craig Livelsberger, Alicia C. McDonald, Joshua E. Muscat

**Affiliations:** 1 Department of Public Health Sciences, Penn State College of Medicine, Hershey, PA 17033, USA; djibrilba@pennstatehealth.psu.edu (D.M.B.); ckayembebandakulu@pennstatehealth.psu.edu (C.K.); lvanscoy@pennstatehealth.psu.edu (L.J.V.S.); akatoch@pennstatehealth.psu.edu (A.K.); yzhang11@pennstatehealth.psu.edu (Y.Z.); clivelsberger@pennstatehealth.psu.edu (C.L.); amcdona_97@comcast.net (A.C.M.); 2 Department of Urology, Penn State College of Medicine, Hershey, PA 17033, USA; jlittlejohn@pennstatehealth.psu.edu; 3 Qualitative and Mixed Methods Core, Penn State College of Medicine, Hershey, PA 17033, USA; evandyke1@pennstatehealth.psu.edu; 4 Pennsylvania Prostate Cancer Coalition, Harrisburg, PA 17120, USA; jimpc2@comcast.net; 5 Center for Survey Research, Penn State Harrisburg, Harrisburg, PA 17057, USA; ncs5161@psu.edu

**Keywords:** prostate, cancer, screening, telehealth, telemedicine, navigation, blacks, men, numeracy

## Abstract

The rapid increase in telehealth has the potential to bring informed decision-making for prostate cancer screening (PCS) at the population level to high-risk individuals. We utilized a global technology platform of electronic health records data repositories (TriNetX) to determine its utility for Navigator-guided decision-making aid for PCS in Black men ages 45–79 years with no history of prostate cancer and PSA testing. Patients from Pennsylvania were invited to participate in a telehealth-delivered informed decision-making session for PCS. Focus groups, social learning theory, visual diagrams, and quantitative data on PCS risks and benefits were used to develop the content of the sessions, which included numerical discussions of risks vs. benefits in Black men. Participants completed several surveys, including baseline demographic and numeracy questionnaires, a one-on-one telehealth session with a trained Navigator, post-Navigation surveys, and an optional follow-up session with a urologist. Eighty-seven participants were consented and recruited. Although the mean numeracy score was only 1.9 out of 6, more than 90% rated as good or excellent that the sessions aided their PCS decision-making skills. This study indicates that Navigation by telehealth offers the ability to assist in informed decision-making for PCS at the population level.

## 1. Introduction

The rapid growth in telemedicine that started in response to the COVID-19 pandemic facilitated access to patient care, including cancer care. The use of telehealth for breast, colon, and cervical cancer screening is also being considered for primary care [1]. Clinical practice guidelines for prostate cancer screening (PCS) describe the use and interpretation of prostate-specific antigen (PSA) tests; however, recommendations for PSA screening as part of routine medical care vary. The US Preventative Services Task Force (USPSTF), for example, did not recommend annual PSA screening for men ages 50–69 years old in 2012 [2] but revised this in 2017 that the decision to undergo PCS screening should be based on discussions with their physicians of the possible benefits and risks [3]. PSA screening remains controversial, as the risk of Prostate Cancer (PCa) varies by population groups, and the risk vs. benefit ratio of screening may not be well understood [4]. The majority of adult men over the age of 40 years surveyed in the Behavioral Risk Factors Surveillance System (72%) reported not engaging in informed decision-making for PCS, even including many who underwent PSA testing [5]. Similar findings were reported specifically for Black men. This number likely represents an underestimate of patient awareness in the population since about 40% of adult men have less than annual physician visits [6].

Non-Hispanic Black men have about twice the incidence and mortality rate of PCa compared with White men [7]. Telehealth or digital health interventions (e.g., information and communication technologies) are potentially an effective means to deliver health care to Black and other high-risk groups. It has been argued that they are also a means for reducing health disparities, especially for Black men, by increasing access to care and reducing social distance between patients and their clinicians [8,9]. Telehealth, therefore, may serve as a wide-reaching technology for delivering decision-making aids to Black men who do not have regular contact with a primary care physician or who do not engage in informed decision-making during physician visits.

Similar conclusions indicate a higher incidence of PCA among Black men in Pennsylvania. A spatiotemporal analysis reveals that although Black individuals constituted only 10% of the total population, they accounted for nearly a quarter of all PCA cases reported in Pennsylvania from 2000 to 2011 [10]. Additionally, another study with the Pennsylvania Cancer Registry shows that Black men had higher overall age-adjusted incidence rates of prostate cancer and more aggressive forms of the disease (400.63 and 153.11 per 100,000 men, respectively) compared with White men from 2004 to 2014 (256.34 and 84.94 per 100,000 men, respectively), regardless of geographical area [11]. Because telehealth can include a wide range of non-clinical services, including education, it does not require physician consultation but can be delivered by other healthcare providers. Patient Navigators can potentially guide men through the process of PSA decision-making. Originally conceived as a delivery intervention for cancer diagnosis and treatment for underserved communities, especially for breast cancer in Black women, it is based on the concept of being a patient-centered healthcare service delivery model that reduces barriers to access to care [12]. Patient Navigation can be a process in preventative care in healthy individuals. Patient Navigation has been used for modifying risk factors such as high body mass index and for smoking counseling and cessation [13].

Patient Navigation may be a model for informed decisions about PCS for Black men. Even physician-delivered decisions for PCS may not be optimal. One study found that physicians have poor familiarity with the USPSTF recommendations [14]. In addition, the empirical benefit/risk ratio of PCS needed to guide such decisions may not be known by the clinical provider or patient. The convergence of shared decision-making through Navigation-based expertise and the recent rapid development, access to, and usage of telehealth may provide the ability to deliver decision-making aid for PCS to Black men on a population basis. The goal of the current project was to test the feasibility of this concept and develop a methodology for Navigation-based informed decision-making using telehealth. Specifically, clinical data repositories of electronic health records allow for the interfacing of men who are eligible for PCS [15].

## 2. Materials and Methods

The Navigation-based program in this project is based on Social Learning Theory (SLT), which assumes that learning about a topic and acting on it is based on observing others, and in particular others who are motivated and provide examples of the knowledge, skills, and self-motivation to make a health decision [16]. SLT provided a foundation for both focus groups and the Navigation project. Systematic reviews show that support from social networks and healthcare providers may facilitate informed decision-making and satisfaction with decisions for undergoing PSA screening [17].

### 2.1. Focus Groups

The purpose of the focus groups was to gather insights and opinions on Black men’s perspective on PCS, their opinions of cancer screening in general, how they perceive health messaging and from who, and to gather their opinion on the use of telehealth for health care and their comfort with technology. This information was then used in the design of the content and delivery of the Patient Navigation-based sessions. 

### 2.2. Focus Group Recruitment

A mail invitation was the main recruiting method utilized by the Penn State Harrisburg Center for Survey Research (CSR). Two lists were purchased from Marketing Systems Group in Horsham, Pennsylvania: (1) a listed residential sample and (2) a cell phone sample. The samples were pulled from the Penn State Health catchment area, which includes Berks, Cumberland, Dauphin, Lancaster, and Lebanon counties, and targeted Black men aged 45 to 65 years. Mail invitations were sent to recruit for this study, in addition to a post on CSR’s Facebook page. Interested participants were directed to contact CSR via toll-free voicemail or email to indicate their interest. CSR then selected participants to ensure a mix of participants with a history and no history of PSA screening in the sessions. After this process, selected participants were contacted and given additional details about the focus group, including the exact location, via email. Reminder emails, texts, and calls were placed the day before each session to confirm attendance.

### 2.3. Focus Group Procedures

Two focus groups were conducted in September 2021 at Penn State Harrisburg, which houses a state-of-the-art facility for focus groups, including audio and video recording technologies. Focus groups were moderated by an executive of the Pennsylvania Prostate Cancer Control Coalition and the American Association of Clinical Research (AACR) leaders in Cancer Advocacy. A total of fourteen individuals participated in two separate focus group sessions. Topics discussed included the use, opinion, and comfort of virtual technologies, use of social media, and knowledge of prostate and prostate cancer prevention, perceptions of inequalities in access to, acceptability, and/or receipt of virtual services such as broadband access. Handouts of prostate-related information were provided, including diagrams of the prostate, PSA testing, digital rectal examination testing, and a heat map of prostate cancer incidence in Pennsylvania showing the excess incidence in Black residents compared with White residents. The sessions were recorded, transcribed, and analyzed using MAXQDA software (Version 2020) for focus group and qualitative analysis. The study team reviewed the analytic findings and worked with the Navigator to determine the best solutions and responses to focus group concerns and used this information to guide the Navigation session. All participants signed a consent form approved by the Institutional Review Board of Penn State University and received compensation for their participation.

### 2.4. Population-Selection for Patient Navigation-Based Intervention

Eligible participants were Black men ages 45+ years with no history of PCa and no history of PSA screening. Eligible participants were identified by TriNetX, a web-enabled technology multi-institutional network of electronic medical records and clinical-based data repositories. TriNetx is managed at Penn State College of Medicine through its Clinical and Translational Science Award (CTSA), funded by the National Center for Advancing Translational Sciences. TriNetX allows for electronic query of electronic health records using filters that can select specific characteristics for the construction of clinical trials and population cohorts. TriNetX is a growing global network that has supported over 19,000 randomized trials and many peer-reviewed publications (1). For the current study, we selected over 1.2 million individual patients seen at Penn State Health from 2019–2021 to identify Black men over the age of 45 with no history of PCA or recent PSA testing. This yielded 3465 individuals. Identifying information, including name, address, and contact information was obtained. Eligible participants were randomly sampled and were contacted by mail using a personally signed invitation letter from the investigation team to participate in this study. Other forms of recruitment were word-of-mouth and StudyFinder, a Penn State platform that matches volunteers to research opportunities at University settings based on participant profiles and study needs. We created a professional studio-based 5-min video presentation of the Navigator, who described this study’s background and goals and provided his contact information. All interested participants were asked to provide a consent form approved by Penn State University’s Institutional Review Board of Penn State University, and compensation was awarded for their participation. Consenting subjects were provided a link to the video to view before the Navigation session. Other materials were provided on the link, including the CDC document “PCS: A decision Guide for African Americans.” They were also helped, upon request, by using links or downloading video apps during the course of this study. 

### 2.5. Navigation Protocol

All aspects of this study were managed by REDCap (Research Electronic Data Capture), a secure, web-based application for databases and online surveys for research that meets HIPAA compliance standards. After consent, participants were texted by REDCAP a link to fill out a baseline demographic survey and a series of short questionnaires from the PhenX Toolkit protocols [18]. 

### 2.6. Baseline Surveys

Baseline surveys included the 1-item California Health Interview Survey Discrimination in Health Care Quality question on whether participants felt their race affected the quality of medical care they receive [19] and the 11-item Health Information National Trends Survey Access to Health Technologies survey [20], which assesses the use of technology such as smartphones, tracking devices, internet, text messaging, electronic medical record portals, and other methods for interfacing with health care or social needs. Other surveys included the 19-item Likert-scaled Medical Outcomes Study (MOS) Social Support Survey on social support [21] and the 6-item General Health Numeracy Test (GHNT-6) that measures basic numerical skills in understanding quantitative health information [22].

### 2.7. Navigation Content

Following the completion of the baseline surveys, subjects were scheduled for a virtual Navigation Session, with the option of follow-up communication with the study Patient Navigator within 30 days. The session lasted approximately 40 min with additional time for questions and answers. The Avaya 2050 IP Softphone was used to facilitate communication between participants and the Navigator who worked remotely. We developed a PowerPoint presentation that incorporated learned lessons from the focus groups. These included perceptions of telehealth for health care, concerns about impersonal communication with unknown healthcare providers, and the role of PSA screening within the general context of cancer screening. 

Social Learning Theory shaped the Navigation session. First, the Navigator for the project was selected based on previous work as a medical advisor in Black men’s health and medical issues. To overcome potential skepticism of telehealth and its impersonal nature as described in the focus groups, the initial part of the session was spent by having the Navigator introduce himself and his background, followed by the participant also giving a brief introduction of himself and his background. Further, to demonstrate an interest in the well-being of the participants, the presentation started with general advice on health care, including the role of tobacco use, physical exercise, nutrition, and alcohol consumption on disease risk. Focus group data indicate that PSA screening should be considered within the context of cancer screening in general. Consequently, materials were prepared that compared and contrasted PSA screening with other cancer screening methods (e.g., colonoscopy). This was followed by a discussion of the prostate, prostate health, and prostate cancer. Focus group findings also indicated skepticism on statistical data in Black men as a motivation for PCS. Consequently, the Navigation session presented quantitative data on the numerical benefits and risks of prostate cancer screening for the latter half of the session (e.g., potential lives saved from PCa per 1000 screened vs. false positives and overdiagnosis. To provide a balanced perspective, we showed the USPTSF recommendations for informed decision-making only for PCS decisions and a YouTube video of a television interview of a physician and a Black prostate cancer survivor recommending annual PSA screening for Black men. Graphs were also presented on the increasing rates of late-stage prostate cancer in Black men, which has occurred since the USPTSF recommendations. The session ended with participants being allowed to ask questions and offer a follow-up session or other communication as needed.

### 2.8. Physician Follow-Up

The Navigator offered the opportunity for participants to schedule a free 20-min session with the study urologist for further questions regarding the clinical management of high PSA scores and prostate cancer. The sessions were informational only and not part of routine care. Session blocks for video conferencing with the study urologists were made available twice a month in the afternoon. Participants were provided a link to register for the available time slots. The physician completed a text-based evaluation of the session content.

### 2.9. Follow-Up Surveys

After completion of the Navigation sessions, participants were automatically sent by RedCap another link to complete a series of follow-up questionnaires. These surveys were the following: the 9-item Agency for Healthcare Research and Quality’s Telehealth Satisfaction Survey [23,24], which was modified for PSA screening, the 9-item Satisfaction with the Interpersonal Relationship with the Navigator [25], a 3-item questionnaire on PSA decision making and plans for a PSA test in the future, and the 16-item Decision Conflict Scale [26], which excluded 3 questions on past-tense decisions, and worded relative to their understanding prostate cancer screening.

For each participant, the Navigator completed a 9-item Likert scale on the perceived participants’ attitude, comfort, and satisfaction with the Navigation session. We imported a Social Vulnerability Score for each participant from Pennsylvania based on the census tract of their residence. The Centers for Disease Control and Prevention/Agency for Toxic Substances and Disease Registry developed a Social Vulnerability Index based on the American Community Survey, which measures socioeconomic status, household characteristics, racial and ethnic composition, and housing and transportation variables (https://www.atsdr.cdc.gov/placeandhealth/svi/documentation/SVI_documentation_2020.html) (accessed on 21 February 2024).

### 2.10. Statistical Methods

#### 2.10.1. Focus Groups

Thematic Analysis. Focus groups were audio-recorded and transcribed verbatim by a professional transcription company (Rev.com). De-identified interview transcripts were reviewed by two experienced qualitative researchers from the Penn State Qualitative and Mixed Methods Core who were neither involved in the design of this study nor in data collection. They used an ontological assumption that is commonly used to explore different realities and perspectives. A postpositivist framework was used to align and orient data with regard to the research question [27]. Analysts first independently reviewed both transcripts and inductively created preliminary categories and codes. The codebook was then discussed amongst the two analysts, definitions for codes were written, and examples of each code were included in the final codebook. The final codebook was then applied to both transcripts using MAXQDA software (Version 2020). Analysts reviewed coding and used Cohen’s Kappa reports to guide calibration and resolution of coding discrepancies between coders. Finally, both coders read the coding reports and created themes that emerged from the patterns in the coding. Attention to qualitative rigor was achieved using standard methods. The neutrality of coding was maintained by using analysts who were not invested in health interventions related to prostate cancer or screening nor researchers who designed this study. Credibility was attended to by including negative perspectives and outlier opinions in the descriptions of findings. Transferability was maintained through the use of rich quotations to support each code and theme. The reliability of coding was measured using Cohen’s kappa, as described above.

#### 2.10.2. Navigation

The mean for continuous variables and the proportion for categorical variables from the baseline and follow-up surveys were calculated using SAS statistical software version 9.4 (SAS Institute, Cary, NC, USA). Graphical presentation of data was used using R version 4.3.3 (https://www.r-project.org/) (accessed on 21 February 2024). Based on the participants’ addresses, we imputed the Centers for Disease Control and Prevention and the Agency for Toxic Substances and Disease Registry Social Vulnerability Index (SVI). SVI is a scale from 0 to 1 that ranks communities according to poverty, lack of access to transportation, and crowded housing. A higher score indicates greater social vulnerability. For this study, the composite SVI score was later partitioned into 4 categories (0.00–0.24 (lowest), 0.25–0.49, 0.50–0.49, 0.75–1.00 (highest)). The 6-item General Health Numeracy Test (GHNT-6) was scored based on the number of items answered correctly. 

## 3. Results

### 3.1. Focus Groups Thematic Analysis

Four themes and several subthemes emerged from the focus group discussion content (Supplemental Materials S1). The main themes were:

Theme 1: While the majority of the participants were comfortable with using technology, the overall perception of telehealth was negative. Specifically, most participants felt that using telehealth was not a preferred modality for healthcare, primarily because of the impersonal nature of the medium. A preference for in-person interaction and social connection were cited as barriers to utilizing telehealth, particularly with regard to medical issues. A few participants framed these negative perspectives around the aspects of medical care that they feel should be part of a visit, including basic physical exam components. Several viewed telehealth as an appropriate solution to care only when seeking treatment for minor medical concerns or issues related to counseling.

These participants noted that the added convenience of telehealth was a reason to overcome the barriers associated with telehealth. Another noted that telehealth was more palatable for mental health services.

Theme 2: Prostate cancer prevalence data were not viewed as an effective strategy to facilitate behavior change; rather, the motivation behind seeking care stems from individual and intrinsic personality factors. This theme emerged out of participants describing what would motivate them to partake in cancer screening. Many did not feel that being informed or educated about statistics related to prostate screening, prevalence, or other empirical evidence would be motivating for them. Rather, they spoke more about how their symptoms or mentality would prompt screening. Others described decisions to seek care or screening as one related to the ‘type of person’ they are or their mentality or intrinsic characteristics.

Theme 3: Participants were aware of the health disparities impacting Black men and believed not seeking preventative prostate cancer care may be a driving factor for such disparities. Many participants had knowledge about the fact that Black men more commonly die from prostate cancer than white men and attributed this to a general state of ‘procrastination’ within the community with regard to screening. In other words, they recognized both the problem (lack of screening) and the implication (increased mortality in Black men). Many attributed this to a strong resistance on the part of Black men to see a doctor. In addition, several participants noted that intrinsic characteristics and personalities played a role in their perceptions that Black men tend not to be screened for prostate cancer.

Theme 4: Participants felt more messaging is needed to reach Black men in order to help improve prostate cancer screening rates. Radio and television spots were commonly mentioned as effective media through which to communicate such messages. A lack of local ‘black radio’ was commonly noted. Many participants recalled hearing ads or service announcements about prostate health while acknowledging that messaging has decreased substantially in recent years.

It was noted that black radio seems a ripe way to increase both knowledge and awareness of the importance of prostate cancer screening.

### 3.2. Navigation Study

#### 3.2.1. Baseline Results

Table 1 shows the basic demographic description of participants including the California Health Interview question on social discrimination and the SVI. Ninety-three participants consented, and 87 participants completed the study protocol from June to December 2023. All but one participant resided in Pennsylvania. The mean (std) age was 58.7 (6.5), 77% were married or living with a partner, and 68% had more than a high school education. All but three subjects stated they had no health insurance. In response to the question of whether they thought race or ethnicity had influenced their past medical care quality, 30% agreed, 30% disagreed, and 40% stated they did not know.

Table 2 shows the baseline Technology Survey related to the use of the Internet during the past 12 months. About 70% reported using smartphones to assist in health decision-making. About 67% reported visiting social network sites such as Facebook or Linked In. About 18% shared information on social networking sites, 7.1% wrote an online diary or blog, 15.3% participated in an online forum or support group for people with a similar health or medical issue, 64.7% watched a health-related video on YouTube, and about 64% have sent or received a text message from a healthcare professional.

Table 3 shows results from the Social Support Survey. About 75% of participants indicated that they received support most or all of the time for each of the individual items.

**Figure 1:**
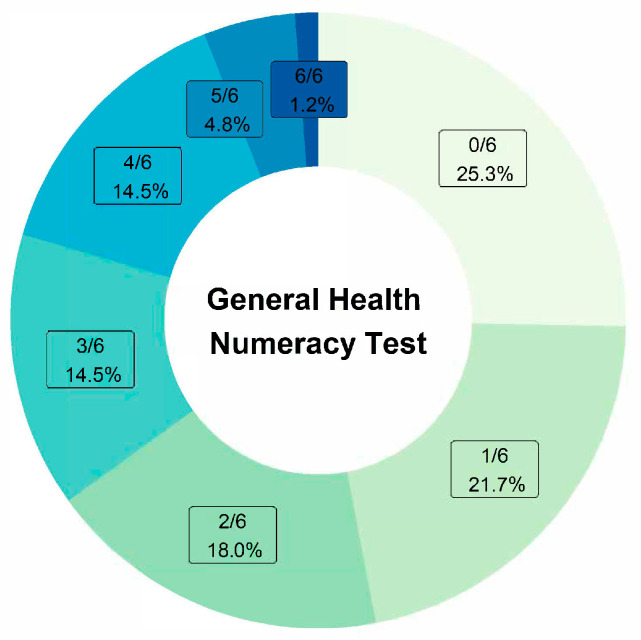
General Health Numeracy Test.

Figure 1 shows the findings of the numeracy test. Thirty-five percent correctly answered half or more of the questions.

There was an inverse correlation between higher levels of social vulnerability (SVI) and higher numeracy score scale (r = −0.18, *p* = 0.11).

#### 3.2.2. Follow-Up Results

##### Telehealth Satisfaction Survey

Supplemental Figure S1 shows the results of the 9-item rating scale for questions on the audio and video quality of the sessions, comfort, ease, and privacy of the sessions, and the Navigator’s ability to schedule the sessions and respond to any technical concerns. About 95% of participants responded as either “good” or “excellent” to these questions.

Supplemental Figure S2 shows the 9-item rating scale for participants’ evaluation of the Navigation session content and the Navigator. About 95% strongly agreed or agreed with questions on session comfort level and adequate time and that the Navigator was courteous, dependable, and paid attention to their needs.

**Figure 2:**
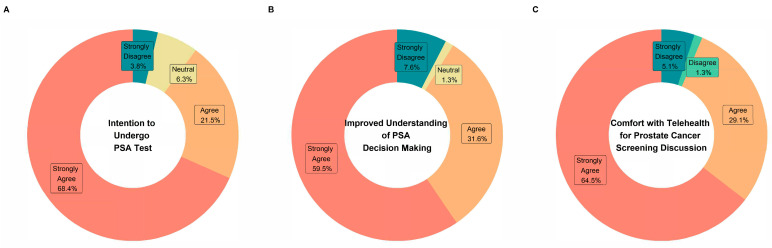
PSA decision questionnaire. (**A**–**C**) are for different questions that are shown in the middle.

Figure 2 shows the responses for the 3-item Decision-Making Questionnaire on PSA. Overall, the respondents provided positive responses to the questionnaire.

##### Decisional Conflict Survey

Supplemental Figure S3 shows the results of this 13-item rating scale applied to PSA screening. The majority of subjects strongly agreed or agreed that they understood the benefits and risks and that they felt confident and informed regarding their choices.

#### 3.2.3. Navigator Evaluation of Sessions

Supplement Table S1 shows the 9-item evaluation by the Navigator on his subjective evaluation of whether the sessions benefitted the participants. On a 5-point Likert scale, the Navigator agreed or strongly agreed that the sessions addressed the participant’s concerns and questions on PCS and that the video sessions went well.

### 3.3. Telehealth Physician Follow-Up Visit

Six participants scheduled an informational session with the study urological surgeon. Topics discussed included the natural history of prostate cancer, treatment options for local and metastatic prostate cancer, and complications of surgical treatment of prostate cancer, including but not limited to erectile dysfunction and urinary incontinence. Patients had the opportunity to discuss topics of their choice in a private doctor–patient, one-on-one environment, which is quite different from the large audience environment typical of a standard prostate cancer patient education forum. This gave them the freedom to openly inquire about their concerns and quandaries without any fear of, or preoccupation with, the judgment of others.

Patients seemed to have clear, well-thought-out questions, and they seemed comfortable and confident about asking them. There were no significant issues regarding technology usage, although there may be some selection bias as this component was optional. Not all original appointments were kept because of scheduling issues on both sides, but patients did not show a strong urgency to reschedule missed appointments.

## 4. Discussion

The current project demonstrates the feasibility of a Navigation-based telehealth model for PCS-informed decisions for Black men. Historically, there have been barriers to prostate cancer screening in the Black community. These include mistrust of the healthcare system, inadequate physician–patient discussions, and not having enough information on PCa risks and treatment options [28]. Interventions to reduce barriers have traditionally been delivered at the church level or community health centers. Based on feedback from Black men, it has been promoted that reducing racial disparities in prostate cancer screening and care should include training providers or peers on educating Black men about prostate cancer [28]. The main message from our own focus groups was that Black men would be more receptive to messages about the different issues of PCS from media sources that appeal to Black listeners and that carried preventive care messages targeting men of color to be checked more often for their prostate health. At the same time, there was a general skepticism about the use of telehealth because it was deemed impersonal. This informed our approach that acceptable telehealth would be more likely to be successful when interfacing with a peer Navigator and physician. In addition, our Navigation session was designed to personalize the session by having the Navigator and participant briefly introduce themselves and discuss general health topics before specifically the issues of PCS. Telehealth offers the opportunity to reduce barriers to screening on a population basis. By utilizing new communication technologies that are now widely available, telehealth offers a number of solutions for increasing informed decision-making. It eliminates the need and cost for transportation to a clinic, especially in more rural areas. It increases access to health care providers. The technology is considered user-friendly even for older individuals who are often less technologically proficient than younger people.

The current project was designed to determine the feasibility of telehealth for PCS in Black men. We utilized a global database and state-of-the-art analytic tools for electronic medical records (TriNetX) to identify Black men with no previous history of prostate cancer and PSA testing and at the recommended age for informed consent. For the current study, eligible participants were identified from Penn State Health, a multi-hospital health system serving patients throughout central Pennsylvania. The use and availability of electronic medical record databases offer some solutions for barriers to PCS. The men sampled from these records already have sought medical care and have demonstrated some trust in the medical system by seeking care either as in-patients or outpatients. The proposed model also builds on our findings from other focus groups that perceived trust, social support, professional prompting, and confidence in decision-making are key aspects of decision-making for PSA screening [17].

Since many men over the age of 40 may not have annual physician visits, and the majority who have visited do not engage in informed decision-making for prostate cancer screening, telehealth by Navigators offers one solution to increase access to decision-making assistance. However, even the process of decision-making for PCS is challenging because providers themselves may not know the risks and benefits, especially for Black men [5]. In 2012, the USPSTF recommended against PSA screening for prostate cancer because the harms were considered greater than the benefits. Since then, diagnostic biopsies and PCa rates have increased, which led to the 2017 recommendations that men under age 70 patients should participate in informed decision-making with their clinicians.

Our study shows similar findings regarding the use of Navigation-based telehealth for cancers other than prostate cancer (PCA). Navigation services have been shown to decrease disparities in colorectal, breast, and cervical cancer screening rates [29]. In a randomized clinical trial providing patient navigation to the intervention group, more patients in the intervention group completed screening compared with the control group for breast cancer (23.4% vs. 16.6%, *p* = 0.009), cervical cancer (14.4% vs. 8.6%, *p* = 0.007), and colorectal cancer (13.7% vs. 7.0%, *p* < 0.001) [30]. Moreover, a systematic review of the use of telehealth for breast cancer management indicates that telehealth can enhance screening rates. It also helps breast cancer survivors manage treatment side effects, alleviate mental distress, and address physical issues related to chemotherapy [31].

Navigation by telehealth has the potential not only to increase the rate of informed decisions but also the necessary content to make an informed choice by educating patients on estimated numerical risks such as overdiagnosis and false positives and benefits (rate of pc deaths prevented by screening). Critically, a patient informed by Navigation may bring expertise to the informed decision-making process that even the care provider may lack. For example, randomized trials on the efficacy of PCS have had too few Blacks to provide race-specific data [32]. By default, recommendations against regular PCS in men under 70 are the same for Blacks and Whites. Our Navigation sessions discussed these issues while also showing newer race-specific data that can assist in the decision-making process. For example, we included published findings that rates of aggressive or late-stage prostate cancer have been rising recently, especially in Blacks, where rates are three times those in Blacks [33,34]. At the same time, the estimated number of lives saved per 1000 screened individuals is considered low, while overdiagnosis is much more common. Even with a general understanding of the actual population and individual risks and benefits, the decision to undergo PSA screening is not straightforward and needs to consider a host of other factors, such as a patient’s personal values, psychological make-up, and allowance for individual clinician’s perspective which may vary from professional organizational recommendations [4]. In our study, participants had the opportunity to discuss these issues with a urologist further. It is likely that future decision-making for PCS will become more complicated as the use of biomarkers and the development of new early detection markers will affect the risks and benefits of screening. Patient education and Navigation may play an important role in these developments and inform decision-making.

Our telehealth model has limitations. We did not have the ability to identify men who do not have any contact with medical care. TriNetX is a database of about 70 million patients worldwide and is growing. This is still a relatively small percentage of all patients, although there are other large, growing Federated Health Data Networks and individual institutions that can also perform population-based outreach programs utilizing electronic medical records. While there are challenges in using electronic health records for clinical research and patient management, the use of demographic information for the purposes of education and patient contact is straightforward. We were able to select those men who had no previous diagnosis of prostate cancer and no medical record of a PSA test. Moreover, another limitation of our study includes the small number of participants in our convenience cohort, the restriction to Black men who were able to participate, and social desirability bias in that those more prone to know about or discuss PCS may be more likely to participate in this research. The findings may not be generalizable and may be subject to the potential for selection bias, as the study participants were volunteers willing to participate. Second, we estimate that our response rate was less than 10%. Some mail was returned to us because of address changes. We did not have multiple mailings or follow-up phone calls with any of the participants. We randomly contacted about a dozen patients by telephone who did not respond to the initial invitation letter mail. Most indicated that they did not recall getting the mail or opening it. A few indicated skepticism that the research incentive was legitimate. A few stated they were not interested. This suggests that we were not able to overcome some of the barriers to screening but that the method of contact could be improved. Future approaches that may be more successful, for example, might include having patient’s personal physicians contact them through an electronic patient portal for a Navigation session before their next clinic visit. It is noteworthy that most participants did not score high on the numeracy test. This indicates that if Navigation is adopted for PCS, the content of the sessions might need to be tailored based on the patient’s numeric skills, as well as other factors like their degree of social support, which varied in our sample. Effective informed consent should be based on understanding the risks and benefits of PCS, and this ability likely varies from patient to patient. While focus groups indicated skepticism on cancer statistics as being a motivating factor for seeking screening, we found that one or two graphical presentations of data were informative and well received within the context of informed decision-making and when discussed by the Navigator. Further work in this area would be to develop and validate Navigation content that accounts for different levels of numeracy skills. Most subjects stated that they had intentions to undergo future PSA screening. However, it was beyond the scope of this study to determine if they were tested after the Navigation sessions. Finally, informed decision-making for PSA testing is only part of the process for potentially managing prostate cancer risk. A single PSA test had a minimal effect on Pca mortality reduction in a clinical trial [35]. Patients with high PSA levels require further care.

The financial model for Telehealth-based Navigation for PCS will need to be determined. The 2024 Medicare Physician Fee Schedule includes a new reimbursement code for patient navigation services for cancer and other diseases and allows for telehealth services for cancer care. Further expansion of insurance-based coverage for PSA screening would facilitate patient access and the ability for informed decision-making.

## 5. Conclusions

Telehealth using Navigation is a method that can facilitate informed decision-making for PCS.

## Figures and Tables

**Table 1:** Baseline demographic characteristics of participants.

Characteristic	No. (%) of Participants *
Age group, years	
46–50	12 (13.95)
51–60	40 (46.51)
61–72	34 (39.53)
Marital Status	
Married	58 (66.67)
Widowed	1 (1.15)
Divorced	6 (6.90)
Separated	2 (2.30)
Never Married	11 (12.64)
Living with a Partner	9 (10.34)
Working Status	
Working Full Time	44 (50.57)
Working Part-Time or Laid Off	4 (4.60)
Looking for Work or Unemployed	6 (6.90)
Retired	11 (12.64)
Disabled	20 (22.99)
Other	2 (2.30)
U.S. Veteran	
No	55 (63.22)
Yes	32 (36.78)
Education level	
12th grade, no diploma	5 (5.75)
High school graduate	15 (17.24)
GED or equivalent	8 (9.20)
More than high school	59 (67.82)
Health Insurance or health care plan	
No	3 (3.45)
Yes	84 (96.55)
Smoking histories	
Never Smoked	52 (60.47)
Former Smoker	21 (24.42)
Currently Smoker	12 (15.12)
California Health Interview Previous Experience of Race/Ethnicity Influencing Medical Care Quality	
Yes	26 (30.23)
No	26 (30.23)
Don’t Know	34 (39.53)
SVI group	
0.00–0.24	15 (17.44)
0.25–0.49	41 (47.67)
0.50–0.74	17 (19.77)
0.75–1.00	13 (15.12)

* The total from each group might not match the overall participant count due to missing data. Abbreviation: SVI: Social Vulnerability Index.

**Table 2:** Baseline Technology Survey.

Questions	No	Yes	
Have you ever used or currently used your smartphone or tablet for the following:			
1. Health Goal Progress Tracker	26 (30.59)	59 (69.41)	
2. Illness Treatment Decision Support	18 (21.18)	67 (78.82)	
3. Healthcare Discussion Assistance	26 (30.59)	59 (69.41)	
4. Non-Tablet/Smartphone Health Monitoring Device Use	38 (44.71)	47 (55.29)	
5. Health Data Sharing with Professional	42 (49.41)	43 (50.59)	
In the past 12 months, have you used the internet for any of the following reasons?			
6. Social Network Site Visit	28 (32.94)	57 (67.06)	
7. Health Information Sharing on Social Media	70 (82.35)	15 (17.65)	
8. Online Diary or Blog Writing	79 (92.94)	6 (7.06)	
9. Participation in Health-Related Online Support Groups	72 (84.71)	13 (15.29)	
10. Watching Health-Related Videos on YouTube	30 (35.29)	55 (64.71)	
11. Text Messaging with Healthcare Professional	31 (36.47)	54 (63.53)	
Questions about your medical records	No	Yes	Don’t Know
12. Healthcare Provider’s Use of Computerized Medical Records	10 (11.76)	2 (2.35)	73 (85.88)
	Not Confident	Somewhat Confident	Very Confident
13. Confidence in Medical Record Safeguards	16 (18.82)	47 (55.29)	22 (25.88)
	No	Yes	
14. Withholding Information Due to Medical Record Privacy Concerns	78 (91.76)	7 (8.23)	

Note: Data for 6 participants are missing for these questions.

**Table 3:** Baseline Medical Outcomes Study Social Isolation Survey.

Questions	None of the Time	A Little of the Time	Some of the Time	Most of the Time	All of the Time
1. Bed Confinement Help Availability	9 (10.47)	2 (2.33)	19 (22.09)	27 (31.40)	29 (33.72)
2. Reliable Listener Availability	2 (2.33)	2 (2.33)	13 (15.12)	29 (33.72)	40 (46.51)
3. Crisis Advice Support	3 (3.49)	2 (2.33)	13 (15.12)	30 (34.88)	38 (44.19)
4. Medical Appointment Assistance	3 (3.49)	5 (5.81)	7 (8.14)	24 (27.91)	47 (54.65)
5. Love and Affection Provider	2 (2.33)	6 (6.98)	4 (4.65)	30 (34.88)	44 (51.16)
6. Enjoyable Companionship Access	2 (2.33)	6 (6.98)	9 (10.47)	34 (39.53)	35 (40.70)
7. Informational Support for Understanding	2 (2.33)	4 (4.65)	13 (15.12)	35 (40.70)	32 (37.21)
8. Personal Problems Confidant	2 (2.33)	4 (4.65)	11 (12.79)	33 (38.37)	36 (41.86)
9. Emotional Support Through Hugs	4 (4.65)	8 (9.30)	9 (10.47)	30 (34.88)	35 (40.70)
10. Companion for Relaxation	3 (3.49)	7 (8.14)	14 (16.28)	28 (32.56)	34 (39.53)
11. Meal Preparation Assistance	6 (6.98)	6 (6.98)	11 (12.79)	27 (31.40)	36 (41.86)
12. Sought-After Advice Source	3 (3.49)	8 (9.30)	18 (20.93)	28 (32.56)	29 (33.72)
13. Mind-Diverting Companionship	2 (2.33)	12 (13.95)	15 (17.44)	30 (34.88)	27 (31.40)
14. Sickness Chore Assistance	5 (5.81)	8 (9.30)	8 (9.30)	31 (36.05)	34 (39.53)
15. Reliable Confidant for Worries and Fears	5 (5.81)	10 (11.63)	7 (8.14)	35 (40.70)	29 (33.72)
16. Personal Problem Advice Source	2 (2.33)	9 (10.47)	14 (16.28)	28 (32.56)	33 (38.37)
17. Enjoyable Activities Companion	2 (2.33)	9 (10.47)	12 (13.95)	31 (36.05)	32 (37.21)
18. Empathetic Listener for Problems	2 (2.33)	10 (11.63)	18 (20.93)	32 (37.21)	24 (27.91)
19. Love and Affirmation Provider	3 (3.49)	8 (9.30)	10 (11.63)	27 (31.40)	38 (44.19)

Note: Data for 5 participants are missing for these questions.

## Data Availability

All relevant data are available from within the manuscript and Supplemental Information Files.

## Supplementary Materials

The following supporting information can be downloaded at: https://www.mdpi.com/article/10.3390/curroncol31070273/s1, Supplementary Material S1: Focus Group discussions and Thematic analysis; Figure S1: Modified Telehealth Satisfaction Survey; Figure S2: Evaluation of the Navigation Session and Navigator; Figure S3: Decisional Conflict Scale; Table S1: Navigator Follow-up Survey on Participants.
