# Supplementary material for: Navigation-Based Telehealth Informed Decision-Making for Prostate Cancer Screening in Black Men
Source: Curr Oncol. 2024 Jun 28;31(7):3698–712. doi: 10.3390/curroncol31070273 (PMC11275421; doi:10.3390/curroncol31070273)
Supplement: Supplementary file 1 [file curroncol-31-00273-s001.zip › curroncol-3027731-supplementary.pdf]

Supplemental File

Index:

Pages 2-6. Focus Group discussions and Thematic analysis

Page 7. Supplemental Figure 1. Modified Telehealth Satisfaction Survey

Page 8. Supplemental Figure 2. Evaluation of the Navigation Session and Navigator

Page 9. Supplemental Figure 3. Decisional Conflict Scale

Page 10. Supplemental Table 1. Navigator Follow-up Survey on Participants

Focus group quotes

Four themes and several subthemes emerged from the focus group analysis.

Theme 1. While the majority of the participants were comfortable with using technology, the overall perception of telehealth was negative. Specifically, most participants felt that using telehealth was not a preferred modality for healthcare, primarily due to the impersonal nature of the medium.

> *"I've done it, I'm just not that fond of it... It's kind of impersonal. I just didn't like it."*

A preference for in-person interaction and social connection were cited as barriers to utilizing telehealth (Subtheme 1A), particularly with regards to medical issues:

> *"Because, you don't, how can you tell me what's wrong with me by talking over the computer screen? If you're going to do that stuff just talk to me over the phone, but I prefer personal contact."*

A few participants framed these negative perspectives around the aspects of medical care that they feel should be part of a visit, including basic physical exam components.

> *"I guess I probably would rather prefer they see me if I have something wrong with me. That way they can check my heart beat, my blood pressure and that type of thing."*

Several viewed telehealth as an appropriate solution to care *only* when seeking treatment for minor medical concerns or issues related to counseling (Subtheme 1B).

> *"Well, I just want to add on, I think telehealth, video conferencing. I think that's excellent if it's something small or minor. I think it's excellent because it does save time again,*

instead of having to go to the doctor's office, add your wait in and they prescribe you aspirin. So I think it's excellent for something small."

These participants noted that the added convenience of telehealth was a reason to overcome the barriers associated with telehealth, however.

"… especially if I got a bad cold and I want to talk to the doctor right now and if he's available and can sit down a few minutes instead of me climbing in the car, trying to find him, yes, I'm more than willing to go pick up my cell phone or get on my computer, "Hey, Doc, help. Oh yes, I'm with that. That's instant help right away."

Another noted that telehealth was more palatable for mental health services.

"I don't mind it for my mental health counseling."

Theme 2: Prostate cancer prevalence data was not viewed as an effective strategy to facilitate behavior change, rather, the motivation behind seeking care stems from individual and intrinsic personality factors. This theme emerged out of participants describing what would motivate them to partake in cancer screening. Many did not feel that being informed or educated about statistics related to prostate screening, prevalence, or other empirical evidence would be motivating for them.

Whether I get statistics or not, I'm going to ask the doctor to be checked. Cause I specifically asked about that. You know, and now, like I said, I see why it's done like that."

Rather, they spoke more about how their symptoms or mentality would prompt screening.

"For me, like in principal, statistics? [No,] that would not have prompted me to go. Cause I had symptoms, it made me go at a younger age, which they recommended at, you

know, back then it was when you turned 50. And I was only in my early forties. So that's what prompted me to go."

Others described decisions to seek care or screening as one related to the 'type of person' they are or their mentality or intrinsic characteristics.

"I mean, I'm one of the persons that if your body, you're not supposed to have any pain in your body, so if you feel some kind of pain I'm going the doctor, period, you know? So that, that's how I feel about it, without statistics."

"I think it's the mentality of someone. I mean, if you've got guys who just want to sit around, just to live, you know, versus someone who likes to work out or be energetic or whatever, you know, me, I like being energetic and working out. So I have no problem."

Theme 3: Participants are aware of the health disparities impacting black men and believe not seeking preventative prostate cancer care may be a driving factor for such disparities. Many participants had knowledge about the fact that black men more commonly die from prostate cancer than white men and attributed this to a general state of 'procrastination' within the community with regards to screening. In other words, they recognized both the problem (lack of screening) and the implication (increased mortality in black men). Many attributed this to a strong resistance on the part of black men to see a doctor.

Participant 1: "I'm just saying, if white guys are at 30%, we could probably be 60 or 70%, because we don't go to the doctor."

Moderator: Mm-hmm. To answer your question, Black die at a rate 2.2 times their white counterpart.

Participant 1: Because we don't go?

Participant 2: And we won't go…

Participant 1: I won't say because we won't go, I feel as though, it's not that you don't go. I think that when we do go,

Participant 2: It's too late."

Similar to Theme 2, several participants noted that intrinsic characteristics and personalities played a role in their perceptions that black men tend not to get screened for prostate cancer.

"And what's so funny about it, is like all the men in my family, they're very secretive about their health. They work good jobs. They get like police officers, and social workers, fully covered. They don't use it, they don't go to the doctors to get checked up and stuff, so that's what I learned when I found that I had prostate disease something just didn't feel right."

Another said simply,

"They sit there and wait till the last minute when it's stage four."

Theme 4: Participants feel more messaging is needed to reach black men in order to help improve prostate cancer screening rates. Radio and television spots were commonly mentioned as effective media through which to communicate such messages. A lack of local 'black radio' was commonly noted.

"But the radio stations I get, I get the ones from Virginia and D.C. because I do them over my phone. So that's Black stations, and they do talk about prostate and whatnot. I would imagine the stations up here don't do it so much directed at Black people."

"Yeah, well we had the Black radio station, you would hear about it more. But now since we don't have a Black radio station, you hardly hear it."

Many participants recalled hearing ads or service announcements about prostate health while acknowledging that the emphasis has fallen by the wayside in more recent years.

> *"You know what, though? That has even slowed down in the past couple of years. At one time, I would say at least 10 years ago, that was on TV all the time, even had infomercials about it. That stuff has calmed down a whole lot now for some reason but 10 years ago, that was big on commercials, people selling prostate pills trying to push them off."*

It was noted that black radio seems a ripe way to increase both knowledge and awareness of the importance of prostate cancer screening.

> *"But I think that needs to be more publicized for men of color to go get checked more often. Should be on the radio and TV and they should make it available.*

Supplemental Figure S1. Modified Telehealth Satisfaction Survey

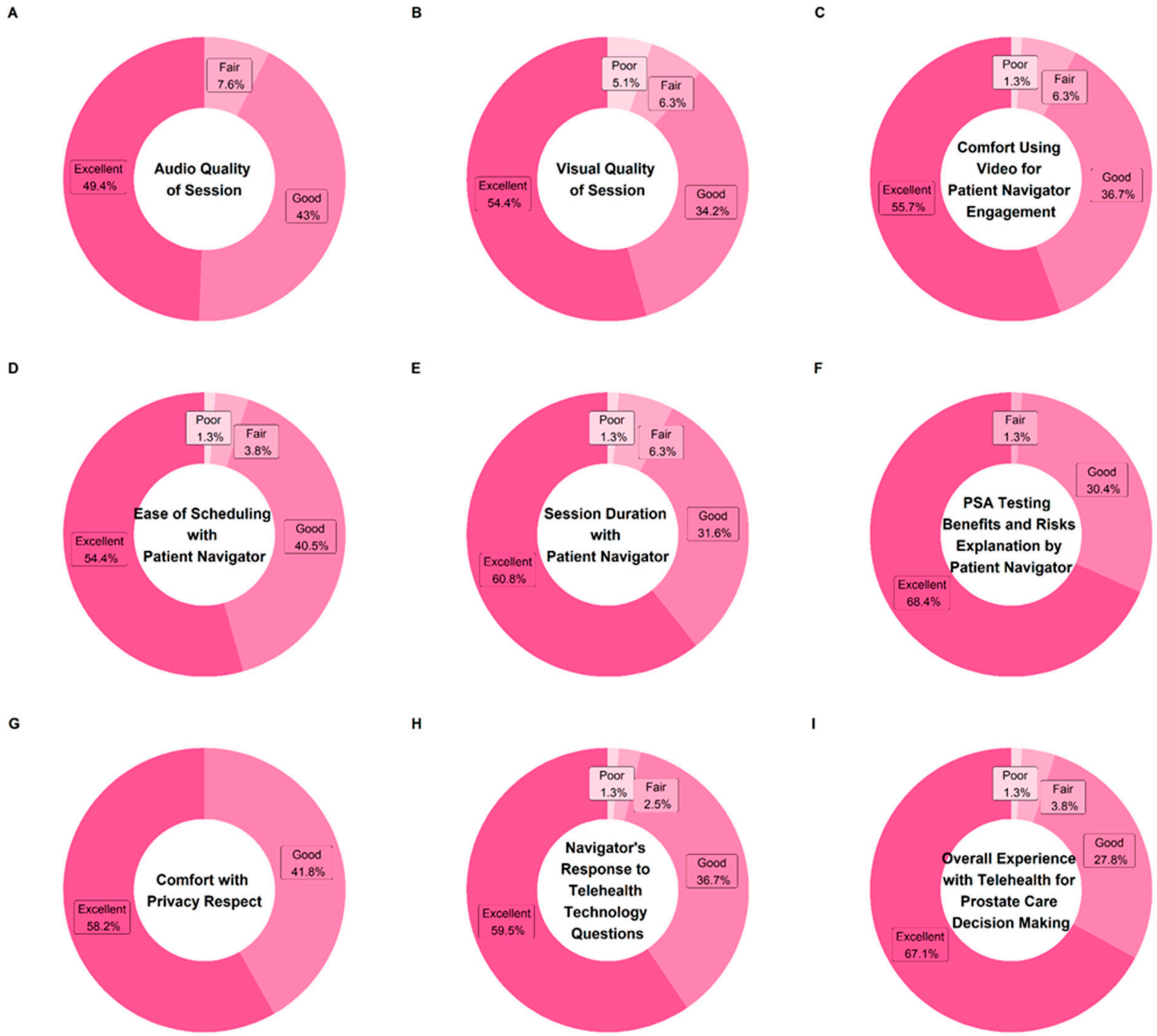

Supplemental Figure S2. Evaluation of the Navigation Session and Navigator

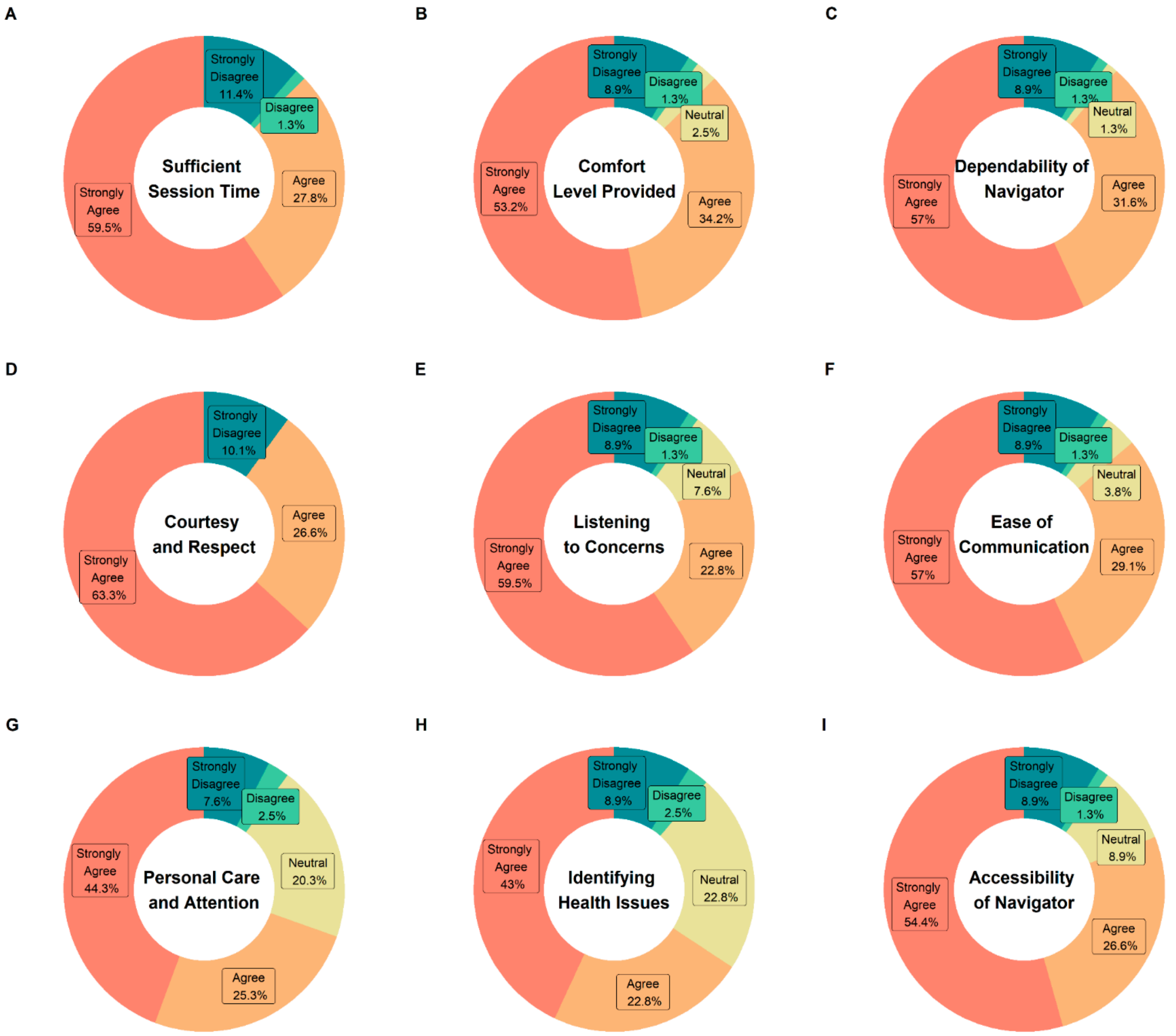

Supplemental Figure 3. Decisional Conflict Scale

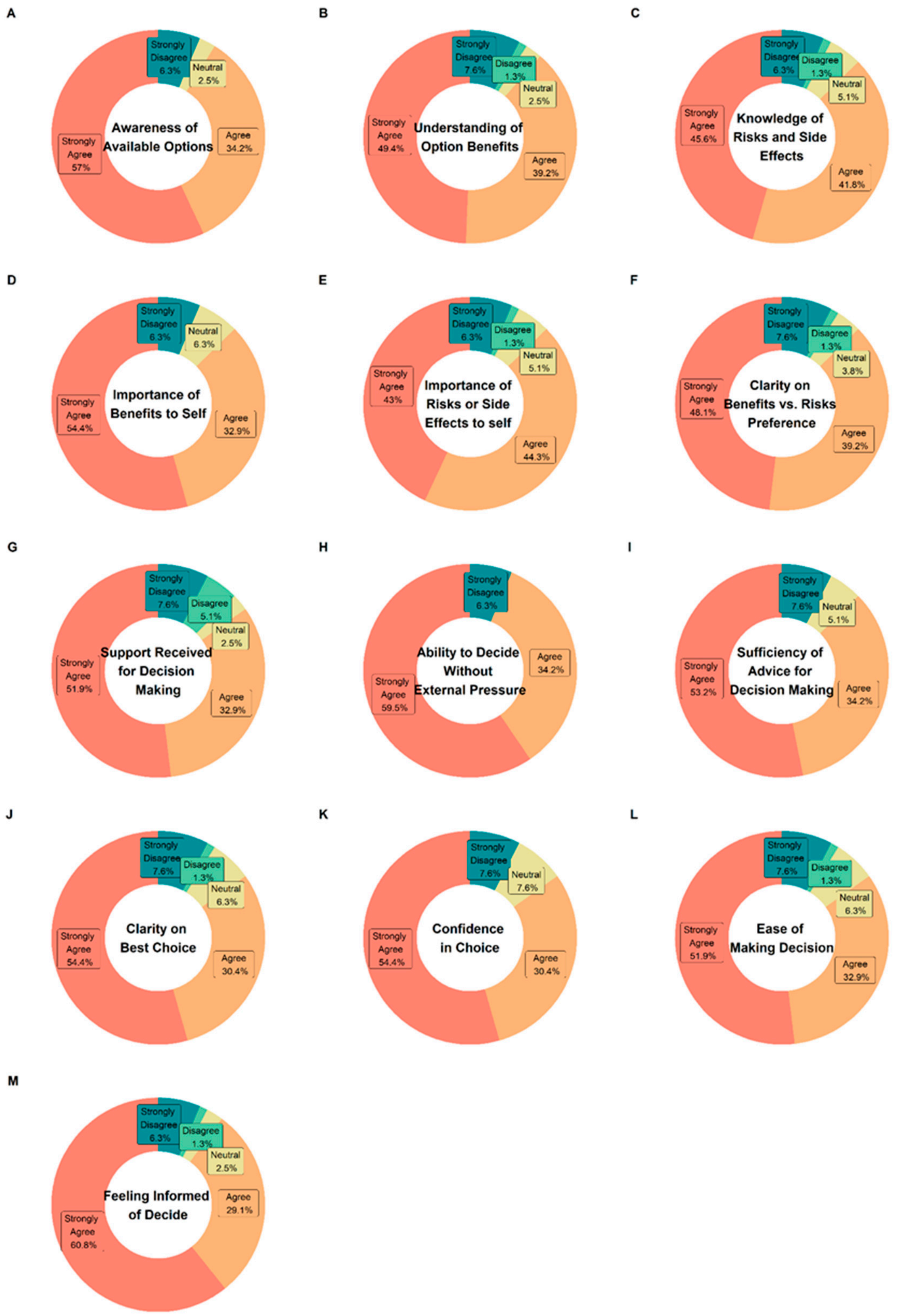

Supplement Table S1 Navigator-Follow-Up Survey on Participants.

Questions	Strongly Disagree	Disagree	Neither agree or disagree	Agree	Strongly agree
1. Addressing Subject's Concerns Effectiveness	0	0	1 (1.27)	44 (55.70)	34 (43.04)
2. Prostate Cancer Screening Knowledge Increase	0	0	1 (1.27)	16 (20.25)	62 (78.48)
3. Increased Enthusiasm for Prostate Cancer Screening	0	0	2 (2.53)	22 (27.85)	55 (69.62)
4. Subject's Courtesy and Respect During Sessions	1 (1.27)	0	0	14 (17.72)	64 (81.01)
5. Positive Subject Response in Navigation Sessions	0	1 (1.27)	2 (2.53)	32 (41.03)	43 (55.13)
6. Ease of Communication with Subject	0	1 (1.27)	2 (2.53)	27 (34.18)	49 (62.03)
7. Subject's Concern for Prostate Health	0	0	0	37 (46.84)	42 (53.16)
8. Identification of Key Issues and Screening Concerns	0	0	2 (2.53)	51 (64.56)	26 (32.91)
9. Subject's Comfort with Video Navigation Sessions	0	0	2 (2.53)	21 (26.58)	56 (70.89)
